# Pott's disease associated with psoas abscess: Case report

**DOI:** 10.1016/j.amsu.2021.103239

**Published:** 2022-01-03

**Authors:** José Paulo Guedes Saint Clair, Írian Evelyn Cordeiro Rabelo, Juan Eduardo Rios Rodriguez, Gustavo Lopes de Castro, Thaís Regina Moreira Printes, Laura Bianca Cabral Fraiji Sposina, Danielle Alcântara Barbosa, Giselle Macedo de Souza

**Affiliations:** a General Surgery Service at Getúlio Vargas University Hospital (HUGV), Avenida Apurinã, 4 - Praça 14 de Janeiro, Manaus, Amazonas, 69020-170, Brazil; b Faculty of Medicine of the Federal University of Amazonas (UFAM), Rua Afonso Pena, 1053, Praça 14 de Janeiro, Manaus, Amazonas, 69020-160, Brazil

**Keywords:** Abscess, Psoas, Spinal tuberculosis

## Abstract

**Introduction:**

Extrapulmonary tuberculosis may develop in any organ system, including the spine. The affection of spinal cord is called Pott's disease and it is the most frequent set. Pott's disease can present as back pain and be related to complications such psoas muscle abscess.

**Case presentation:**

We report a case of 23-year-old Brazilian woman, natural from Amazonas, presenting with psoas abscess associated to Pott's disease treated with surgical debridement and drainage and extended tuberculosis scheme.

**Discussion:**

Psoas abscess is rare and the main agents related to psoas abscess are *Staphylococcus aureus*, followed by *Streptococcus* and *Escherichia coli*. Retrofascial abscesses usually originate from bone lesions, including spine tuberculosis as our case, or due to contiguity of the retroperitoneal space.

**Conclusion:**

Psoas abscess related to Pott's disease is a difficult diagnosis, requiring high suspicious and proper investigation through good anamnesis, CT scan and culture.

## Highlights

• Extrapulmonary tuberculosis may develop in any organ system, including the spine.
• Psoas abscess is rare and the main agent related to psoas abscess is *S. aureus.*
• Treatment of Pott's disease depends of early diagnosis and antituberculous therapy.

## 1 Introduction

Extrapulmonary tuberculosis may involve in any organ system, including the spine. Spinal tuberculosis or Pott's disease is one of the osteoarticular tuberculosis types, alongside with arthritis and osteomyelitis. Pott's disease is the most frequent and its clinical presentation is variable and depends on which stage disease is [[1], [2], [3], [4]].

Psoas abscess, first described by Mynter in 1881, is an uncommon disease and relevant infectious pathology due to its morbimortality. Psoas muscle originates from the transverse processes of the 12th thoracic and all lumbar vertebrae. Its incidence has grown with imaging exams technology advance, facilitating diagnosis. Psoas abscess can be divided into primary and secondary based on the presence or absent of previous condition. Gram-negative bacteria are the main microorganisms involved in pathophysiology, such as *Escherichia coli*, *S. aureus* and *M. tuberculosis* [5,6].

Treatment success of Pott's disease depends on early diagnosis and antituberculous therapy and, in some cases, surgical drainage. The last therapeutic option is more often associated with psoas abscess presence [7].

We report a case of 23-year-old Brazilian woman, natural from Amazonas, presenting with psoas abscess associated to Pott's disease treated with surgical debridement and drainage and extended tuberculosis scheme according to the Ministry of Health. This case report is being reported in line with the SCARE 2020 criteria [8].

## 2 Case presentation

A 23-year-old Brazilian woman, brown color, natural and proceeding from Manaus, Amazonas, with chief complaint of tumor-like mass with progressive growth in left lumbar spine for three months. She also complained about relevant local hyperemia and pain, worsened while sitting. The patient also reported dry sporadic cough, especially during night and pneumonia treatment about a year ago. No fever or weight loss history was reported, as well as no drinking, intravenous drug use, contact with tuberculosis, previous surgery or comorbidities.

In general physical exam, a paravertebral bulging in left lumbar region was noticed at L3 – L5 level, with cystic consistence in palpation without any secretion. A soft tissue ultrasonography requested in ambulatory environment revealed a septate cystic image with heterogeneous content, low amplitude echoes and low intensity periferical vascularization, measuring 13 x 5.9 × 12.9 cm with volume of 521.9 mL, around 0.75 cm from skin surface.

**Fig. 1:**
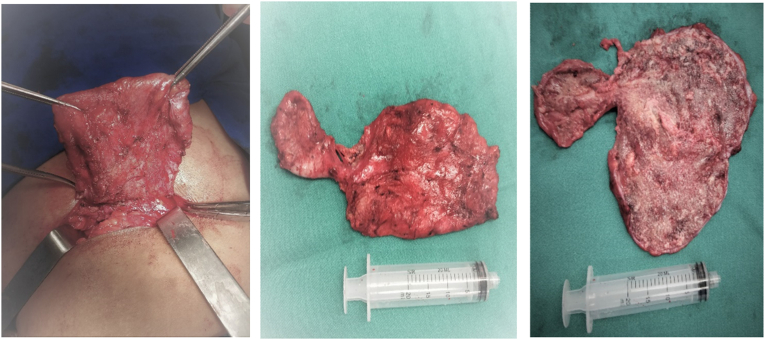
A. Cystic lesion excision under local anesthesia. B. Macroscopic aspect of the excised lesion. C. Intern macroscopic aspect of the excised lesion.

An excisional biopsy of the cystic lesion with thickened walls was performed, noticing cheesy spots and adherence with muscle plan with drainage of seropurulent liquid in big amount (approximately 500 mL) and a path with vertebral region was identified (Fig. 1). The excised material was sent to histopathological analysis.

The patient returned to general surgery ambulatory after four months with histopathological results and presenting bulging recurrence in the operatory wound area associated with odorless seropurulent liquid drainage. No other symptoms were complained. The histopathological analysis revealed in macroscopy, a mass with fibroelastic consistence and brown-like color. In microscopy, thickening of conjunctive tissue with fibrosis and collagen, inflammatory infiltration with lymphocytes, plasma cells, epithelioid cells and some giant cells were identified.

**Fig. 2:**
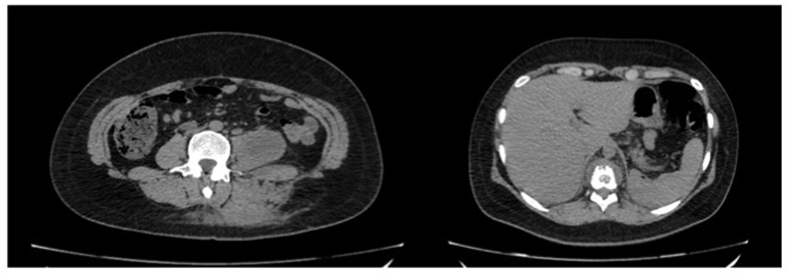
A. Hypodense image in left psoas suggesting collection (red arrow). B. Litics alterations around D 11 vertebral body (red arrow).

Faced this result, the patient was admitted in our hospital and an abdominal computed tomography (CT) without intravenous contrast was requested. CT showed long hypodense image with delimited walls in left psoas muscle, measuring 10 x 4,2 x 4,5 cm (98 cm^3^), unspecific without further investigation with contrast study (Fig. 2). Retroperitoneal lymphnodes were increased in number, measuring up to 0,5 cm. Other tomographic findings were litic alterations of D 11 and D 12 vertebral bodies in association with paravertebral soft tissue raise corresponding to communication with psoas muscle collection in L 3 to L 5 level and measures of 8,1 x 2,2 x 4,8 cm (45 cm^3^). This collection presented extension to cutaneous surface, corresponding to fistulous path.

Research for acid-alcohol-fast-bacilli (AAFB) in secretion was positive in Ziehl-Neelsen color process and 01 AAFB was found in analysis. There was no pathogen growth in secretion culture required during hospitalization. It is important to mention that the histopathological finding is not compatible with a diagnosis of tuberculosis. However, we believe that the patient developed tuberculosis only after the first procedure.

Due to CT findings suggestive of spinal tuberculosis and secretion positive baciloscopy, the patient was submitted to surgical debridement with psoas muscle drainage by general surgeons, as also prescribed extended therapeutic scheme for osteoarticular tuberculosis according to recommendations of the Ministry of Health.

The patient was discharged five days after surgery and referred to specialized service of infectology, pneumology and orthopedics. In follow-up, she presented fully recovery of affected region without recurrence until the present moment.

## 3 Discussion

Although abscess is a common surgical lesion, unusual findings underlying abscesses are not uncommon^9,10^. Psoas abscess is classified as primary or secondary. The first one is more common in tropical and in development countries while the second one can be monomicrobial or polymicrobial [4,5,[9], [10], [11]].

Primary occurs as result of hematogenic or linfatic dissemination and tend to affect children or young adults. Risk factors include diabetes, intravenous drug use, HIV infection, renal insufficiency and other forms of immunosuppression. Secondary abscess develops directly from a psoas muscle infection propagation in an adjacent structure. Risk factors in this case include trauma in inguinal, hips or spine region [4,5,11].

Main agents related to psoas abscess in Brazil are *Staphylococcus aureus*, followed by *Streptococcus* and *Escherichia coli*. *Mycobacterium tuberculosis* is a frequent bacterium in the psoas abscess in regions with high prevalence of tuberculosis, although is rare in the United States [5,12]. Another important factor is that the northern region of Brazil has the highest rate of hospitalizations for tuberculosis and the highest mortality rate in the country (2.9 deaths/100,000 inhabitants) [13].

Although our patient is a woman, psoas abscess is more common in men under 20 years of age. Other contradictory information with our case is that psoas abscess are more frequent in right side, but no explanations about this fact have been described in the literature [12].

Signs and symptoms in patients with psoas abscess may present as back or flank pain, mass-like formation, fever, anorexia and weight loss. The pain is present in 91% of the cases, followed by fever in 75%. Symptoms can also be unspecific and extend for weeks to six months in subacute state. These symptoms have non-specific features which can often lead to a late diagnosis. In one study, the average time between symptom onset and diagnosis was 22 days, while the longest hiatus was 42 days for one third of the patients [4,11].

Tuberculosis (TB) in the spine is an old condition and usually affects inferior thoracic and superior lumbar regions, while cervical and superior thoracic regions are less commonly involved. The most common site of extrapulmonary involvement is skeletal, with 50% being spinal affection. In general, Pott's disease causes inflammation process in the intervertebral joints and can lead to spinal cord compression. In developed countries with more access to imaging studies, Pott's disease may be identified earlier as vertebral osteomyelitis with local complications, such as psoas abscess [14,15].

Retrofascial abscesses usually originate from bone lesions, including spine tuberculosis as our case, or due to contiguity of the retroperitoneal space, especially in colonic lesions. Other etiologies described in the literature are suppurative iliac adenitis, renal rupture, ureterolithiasis, pleural empyema, metastasis and trauma [16].

Psoas abscess diagnosis can be suspicious for clinical insights and confirmed with imaging investigation. Gold standard for imaging diagnosis is magnetic resonance (MR) with biopsy guided by computed tomography (CT). CT is the ideal radiographic modality to evaluate psoas abscess, although its sensibility has been limited in the first stage of the inflammatory process. Etiological organism identification requires culture of the blood, pus or in rare cases, tissue surgically excised [4,11,15].

CT findings include focal hypodense image, local fat infiltration and presence of gas or air fluid in the muscle. MR may allow better definition of soft tissue and adjacent structures, such as vertebral bodies. Simple abdominal radiography suggests definition loss of the psoas muscle, abnormal soft tissue shadows and presence of gas, however these findings are not consistent or definitive [4,11].

Microscopy and culture of infected material are recommended for the diagnosis of spinal TB [15]. In our case, the histopathological analysis revealed thickening of conjunctive tissue with fibrosis and collagen, inflammatory infiltration with lymphocytes, plasma cells, epithelioid cells and some giant cells, a finding that makes us think about the possibility of tuberculosis infection after the biopsy. Acid-alcohol-resistant bacilli was detected in the abscess secretion analysis.

There are several differential diagnoses of Pott's disease, which can be responsible for delay in diagnosis, such vascular alterations, degenerative disorders of the spine and also genetic and traumatic conditions. Two of the most important are Langherhans cell histiocytosis in pediatric patients and neoplasms in patients over 60 years of age [17]. Differential diagnosis for psoas abscess may include: (1) muscle psoas hematoma, more frequent in anticoagulation or hemorrhagic disturb cases, with clinical similarity but radiological findings distinguished, (2) hip septic arthritis and metastatic conditions, such as mucinous adenocarcinoma. Calcium pyrophosphate and herniated disc fragments deposition can mimic psoas muscle abscess as well [11].

Psoas abscess treatment consists in drainage and immediate broad spectrum antibiotic therapy. The antituberculous therapy for skeletal tuberculosis is the same for pulmonary tuberculosis, nevertheless therapeutic scheme can be modified due to drug interaction in cases of HIV coinfection or antibiotic resistance. Treatment duration for muscle-skeletal tuberculosis is uncertain. For most patients, six to nine months of therapy are enough, although an extended treatment (9–12 months) is opted for schemes that do not include rifampicin or patients with extensive or advanced disease, especially in cases where it is hard to evaluate therapeutic response [11,15,18].

Complications of Pott's disease are related to late manifestations, such as spinal cord compression or destruction of the vertebras, while psoas abscess incorporates septic shock (20% of the cases), deep venous thrombosis (due to extrinsic compression of iliac and femoral veins), hip septic arthritis, hydronephrosis (due to ureteral compression) and paralytic ileus [15,18].

## 4 Conclusion

Pott's disease is a great diagnosis challenge, especially because there are no evidences of thoracic active disease in most cases. Thus, it is an underdiagnosed disease and usually with late approach. Breathing symptoms associated with patients diagnosed with abscesses and chronic back pain must also be investigated, regarding the differential diagnosis including Pott's disease. In the present case, etiological identification associated to the abscess was essential to ensure adequate therapeutic management and by consequence, great clinic evolution of the patient, preventing from recurrence of abscess formation and other unnecessary approaches.

## Patient perspective

The patient reported in the follow-up he was grateful for the problem solution after surgery and that he could go back to his teaching activities without any of previous difficulties.

## Ethical approval

This study was exempt from ethnical approval.

## Sources of funding

We did not receive funding from any source.

## Author contribution

José Saint’Clair, Juan Rodriguez, Gustavo de Castro made contributions to conception and design. Ìrian Rabelo, Thais Printes, Juan Rodriguez and Gustavo de Castro collected the patient details and wrote the paper. Giselle de Souza, Laura Fraiji made contributions to patient management. José Saint’Clair, Danielle Barbosa critically revised the article. All authors read and approved the final manuscript.

## Consent

Written informed consent was obtained from the patient for publication of this case report and accompanying images. A copy of the written consent is available for review by the Editor-in-Chief of this journal on request.

## Registration of research studies

1. Name of the registry:

2. Unique Identifying number or registration ID:

3. Hyperlink to your specific registration (must be publicly accessible and will be checked):

## Guarantor

José Paulo Guedes Saint’Clair.

## Provenance and peer review

Not commissioned, externally peer-reviewed.

## Declaration of competing interest

All authors declare no conflict of interests.
